# Application of Deep Convolution Network Algorithm in Sports Video Hot Spot Detection

**DOI:** 10.3389/fnbot.2022.829445

**Published:** 2022-05-26

**Authors:** Yaling Zhang, Huan Tang, Fateh Zereg, Dekai Xu

**Affiliations:** ^1^School of Management, Beijing Sport University, Beijing, China; ^2^College of Sports, Leisure and Tourism, Beijing Sport University, Beijing, China; ^3^Department of Theory and Methodology of Football, Chengdu Sport University, Chengdu, China; ^4^Institute of Physical Education, Hoseo University, Asan-si, South Korea

**Keywords:** deep convolutional neural network, human motion recognition model, big data technology, hot spot detection, sports video

## Abstract

Sports videos are blowing up over the internet with enriching material life and the higher pursuit of spiritual life of people. Thus, automatically identifying and detecting helpful information from videos have arisen as a relatively novel research direction. Accordingly, the present work proposes a Human Pose Estimation (HPE) model to automatically classify sports videos and detect hot spots in videos to solve the deficiency of traditional algorithms. Firstly, Deep Learning (DL) is introduced. Then, amounts of human motion features are extracted by the Region Proposal Network (RPN). Next, an HPE model is implemented based on Deep Convolutional Neural Network (DCNN). Finally, the HPE model is applied to motion recognition and video classification in sports videos. The research findings corroborate that an effective and accurate HPE model can be implemented using the DCNN to recognize and classify videos effectively. Meanwhile, Big Data Technology (BDT) is applied to count the playing amounts of various sports videos. It is convinced that the HPE model based on DCNN can effectively and accurately classify the sports videos and then provide a basis for the following statistics of various sports videos by BDT. Finally, a new outlook is proposed to apply new technology in the entertainment industry.

## Introduction

Today, the Chinese are enjoying an ever affluent material life as the domestic economy boosts, with which their pursuit for spiritual wellbeing rises sharply. Especially with the increasingly mature computer and networking technology (CNT), online resources have become primary resort of people for spiritual pursuit and substantially enhance entertainment industry of China. Thus, it is not uncommon for Chinese citizens to watch live sports broadcasts or sports videos to escape the fast-paced and competitive study, life, and work.

The deep convolutional neural network (DCNN) algorithm is particularly apt for image recognition (IR). Once, feature extraction (FE) has hindered the further development of IR techniques, given the complexity of image data over other common data types, such as texts. At that time, image features were mainly represented through artificial means that are based on understanding or estimation of researchers. Such a situation has not seen a fundamental change until the emergence of CNN, almost tailor-made for automatic FE for images over traditional machine learning (ML). Thus, CNN has dramatically simplified many emerging technologies, such as IR and Pattern Recognition (PR) (Sulam et al., 2019; Maiorino et al., 2021). In particular, big data technology (BDT) uses Big Data to provide analytical solutions to complex practical problems. The so-called big data refers to a collection of structured, semistructured, and unstructured data sets that are so voluminous for conventional data processing algorithms to tackle appropriately that include the acquisition, storage, management, and analysis of big data. Big data features massive scale, rapid flow, diverse types, and low-value density (Romanowski, 2019; Tolan et al., 2020).

In the Big data era, deep learning (DL) has become one of the most favored technologies in various industrial applications, on which extensive research has been conducted. For example, Singh et al. (2021) proposed a novel pointwise convolution design using spatial input information. Specifically, they extracted and refined the input spatial context information on two scales (Singh et al., 2021). Finally, a time-series feature vector for classification trained the support vector machine (SVM). Sandula et al. (2021) constructed a new camera motion classification framework based on the hue-saturation-intensity (HSI) model to compress block motion vectors. The designed framework sends the input to the inter-frame block motion vector decoded by the compressed stream to estimate its size and direction and assign the motion vector direction to hue and the motion vector size to saturation under a fixed Intensity. Then, the HSI distribution was converted to Red, Green, Blue (RGB) images. After that, CNN was used for supervised learning to identify 11 camera motion modes that include seven pure camera motion modes and four hybrid camera modes. The results showed that the recognition accuracy of this method for 11 camera modes reached over 98% (Sandula et al., 2021). Rajesh and Muralidhara (2021) designed a reconstruction loss based on new driving and used the implicit multivariate Markov random field regularization method to enhance local details. They used a multi-column network to propagate the local and global information from the context to the target coated area (Rajesh and Muralidhara, 2021). Churchill et al. (2020) applied the original data from a single diagnosis (the electron cyclotron emission-oriented imaging diagnostic data) from the Tokamak. They designed a neural network (NN) architecture for the prevalent interruption prediction problem: the fusion energy Tokamak (Churchill et al., 2020). Jamali et al. (2021) built a timeliness and computational efficiency CNN structure and combined it with other mainstream NN and ML algorithms: random forest (RF), Gaussian network, and Bayesian optimization Tree. As a result, the training time was significantly shortened (Jamali et al., 2021). Chen et al. (2020) employed DL in video recognition and put forward a DCNN model for human motion recognition (HMR) (Chen et al., 2020). Minhas et al. (2019) devised a track and field video-oriented shot classification method using an eight-layer AlexNet, including five convolution layers and three fully connected layers. The shot was divided into a long shot, medium shot, close-up shot, and off-site shot. The standardization and screening layer response on the feature map improved overall training and validation performance on different databases (Minhas et al., 2019). Ramesh and Mahesh (2020) analyzed the performance of the pre-training network, used the AlexNet for FE, image classification (IC), and transfer learning (TL), and compared its performance with the DCNN with a user-defined layer on the same dataset. Consequently, the performance of DCNN was improved (Ramesh and Mahesh, 2020). To sum up, DCNN has been applied in various aspects, the most important of which is used for data processing and machine vision recognition, which can greatly improve the accuracy of HMR in the video. In particular, AlexNet has gained enormous technological advances through the research of experts and scholars on CNN technology in different fields. Generally, an AlexNet is a CNN structure with five convolution layers and three fully connected layers, with two parallel graphics processing units (GPUs) for computation acceleration. Meanwhile, the upper and lower information can interact at the third convolution layer and fully connected layer. AlexNet extends the basic principles of CNN to deeper and wider networks. Concerning the current research topic, the sports video's hotspot detection, it is found that different types of CNNs generate great differences in prediction accuracy. Therefore, the present work will optimize the hotspot detection model combined with BDT to empower the scoring system effectively.

Thereupon, the present work proposes a human pose estimation (HPE) for sports video classification, recognition, and detection using DCNN and BDT. The available relevant literature mostly uses the DCNN algorithm to extract the features of sports images and can correctly identify the character poses, only with relatively low recognition accuracy. By comparison, the present work innovatively employs a Haar-like FE algorithm for image data screening and enhancement, thus dramatically improving the accuracy of HPE, reducing video delay, and enhancing the efficiency of athlete motion capture. Thus, a sports video recognition and classification model based on BDT and DCNN is implemented, and the simulation experiment is carried out. The innovation lies in optimizing the traditional NN model by combining BDT with DCNN. Afterward, by comparing single HPE algorithms and fusion models, the most appropriate combination is selected to implement the proposed athlete-oriented HPE model, increasing video pose recognition accuracy and efficiency. As a result, a new perspective is proposed for computer technology (CT) to promote the development of the entertainment industry. Innovatively, the first-identification-and-then-recognition method is employed to address the HMR problem in sports video sequences. The target i algorithm (TIA) is primarily used to detect poses of athletes in sports videos. Then, the TIA is used to identify category of athletes of motion behaviors in the video sequence. The value and excellence of present research provide a more effective method to detect the pose of the athletes in sports video and offer a new outlook for the related research of high-precision HPE in the future.

## Construction of Sports Video Classification and Recognition System

### Big Data Technology

**Figure 1:**
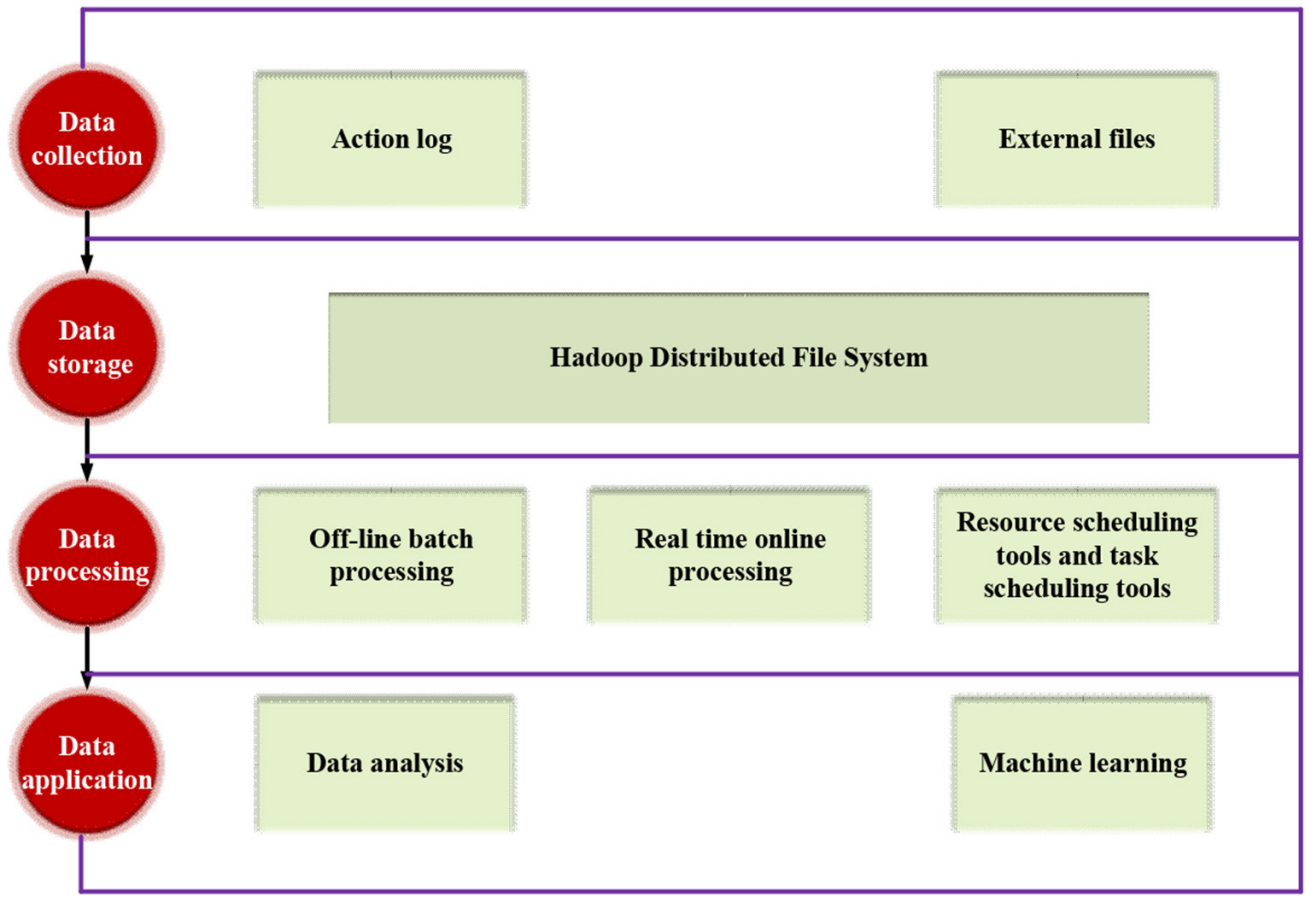
Basic architecture of Big Data.

Big data refers to extra-large-scale datasets that cannot be captured, managed, and processed by conventional software tools within finite iterations. It is a voluminous, high-growth-rated, diversified information asset that requires a new processing mode with more robust decision-making (DM) power, insight and discovery power, and process optimization ability (Singh et al., 2019). Figure 1 shows the basic architecture of Big Data.

Generally speaking, before the final data report review or data-based algorithm prediction, BDT will go through the following processing links: data collection, data storage, data processing, and data application.

Here, the primary sources of experimental data are (a) background data of sports videos from major online platforms, (b) rating data of various television (TV) sports channels, and (c) viewing data of various broadcast channels during major sports events (Opotowsky and Webb, 2020; Saju and Ravimaran, 2020).

### Image Classification and FE

**Figure 2:**
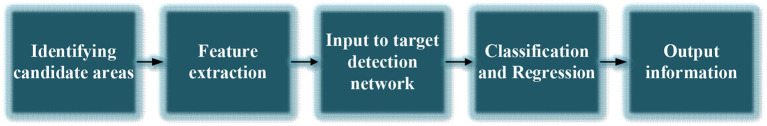
Detection flow of the target identification algorithm (TIA).

Figure 2 signifies the flow of an ordinary TIA.

#### Select the Bounding Box

When such target information as the number, size, and type in a picture is unknown, it is necessary to ensure two aspects of the algorithm design: (a) the bounding boxes for FE include as many items to be inspected as possible and (b) the bounding boxes for FE are as few as possible (Chavez-Badiola et al., 2020).

**Figure 3:**
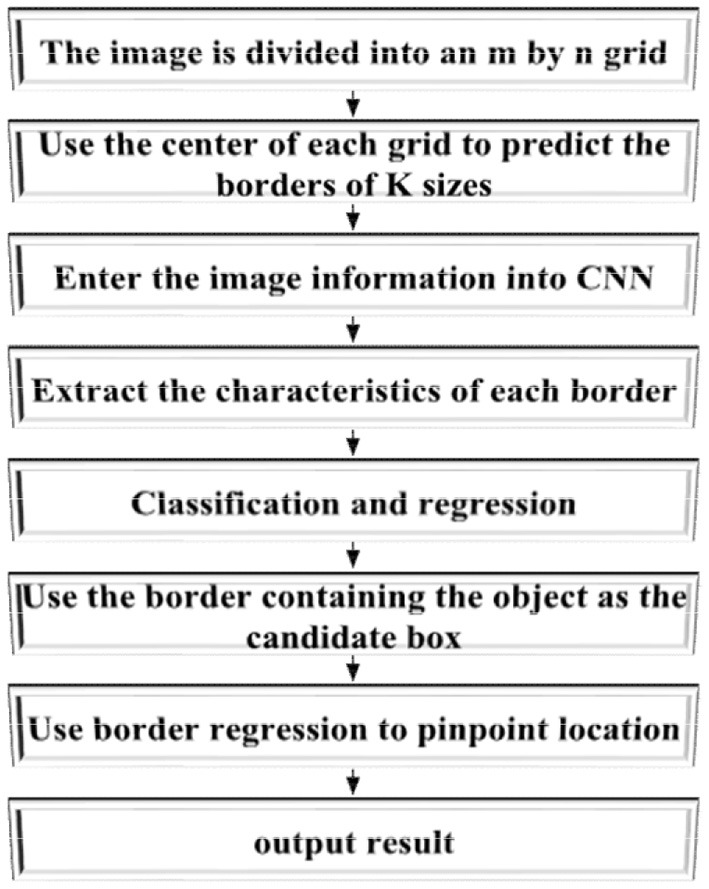
Bounding box selection by Region Proposal Network (RPN) method.

Three methods are available for selecting bounding boxes: Sliding Window Algorithm (SWA), Selective Search (SS), and Region Proposal Network (RPN). SWA reflects the detailed features of images in the sliding window. Global FE methods mainly include principal component analysis (PCA), gray level-gradient co-occurrence matrix (GLCM), frequency domain analysis method, scale-invariant feature transform (SIFT), and Haar-like features. Remarkably, the present experiment chooses the RPN method for bounding box selection with the following implementation steps. First, pictures are used as initial input for SS, according to which the bounding boxes are further determined. Then, the pictures are segmented into smaller images with unique features, similarities, and gradients used for splicing the bounding boxes into large blocks. Finally, the size of bounding boxes is determined according to the blocks. The RPN method reduces bounding box extraction from images when compared with the SWA. Meanwhile, RPN uses the CNN algorithm to extract bounding boxes and generates dense boxes by a fixed scale. Further, CNN can classify and point out the positions of the boxes so that the final bounding boxes could contain all objects, thus minimizing the number of bounding boxes (Abu Hasan et al., 2020; Budiman and Sugiarto, 2020; Tarsitano et al., 2021). Figure 3 illustrates the RPN algorithm flow.

#### Feature Extraction

Feature Extraction technology is the core of computer vision and is the basis of IR and IC. FE involves both bottom-layer features (image color and texture) and advanced features (including image generalization and abstraction). Information, such as size and quantity, helps in visualizing asset management (Khaleefah et al., 2020; Saad and Hirakawa, 2020). These features are listed as follows.

- Image color: A coordinate system can be established based on the RGB three-hue format and calculation. RGB can adjust the vector size to express different image colors as vector components. RGB three-hue format can create color distribution histogram, color space coding, and image hash features.
- Image texture refers to the distribution of image hue and light intensity in a certain area, including the GLCM.
- Image edge: It summarizes the outline of image content. Standard FE algorithms include Canny or Sobel operator edge detection.
- Image transformation: The time-to-frequency domain transformation can depict the image details, where the statistical and content features are separated.
- Image depth: The CNN algorithm is used for post-extraction of standard and deep image features, widely used to describe image features at the semantic and bottom feature levels.

#### Feature Matching

Correlation between image features can be different. The proposed algorithm calculates the image correlation by defining the distance between two features (Gururaj and Tunga, 2020; Sugiarto et al., 2020; Desai et al., 2021), as detailed below:

- Euclidean Distance (ED): Equation (1) counts the distance (ED) between two feature vectors in the Euclidean space.

(1) $$\begin{array}{l} d_{xy}=\sqrt{\sum_{k=1}^{K}{\left(x_{k}-y_{k}\right)}^{2}} \end{array}$$

**Figure 4:**
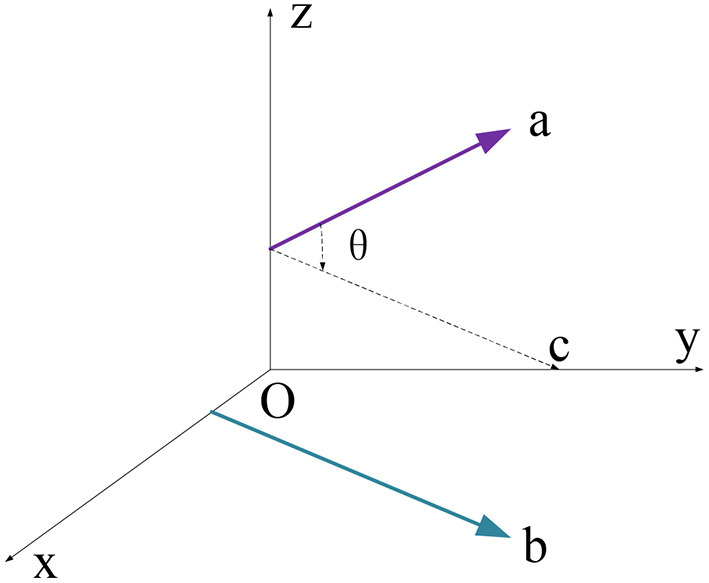
Schematic diagram of the cross product.

In Equation (1), *x* and *y* are all feature vectors, *K* stands for the feature vector dimension, and *d*_*xy*_ refers to the ED. The larger the *d*_*xy*_ is, the larger the correlation between two points and vice versa.

b. Cross product. This parameter describes the consistency of vector direction and value in a different dimension, expressed as Equation (2) and displayed in Figure 4.

(2) $$\begin{array}{l} |\vec{a}\times \vec{b}|=|\vec{a}|▪|\vec{b}|▪\sin\theta \end{array}$$

Where $\vec{a}$ and $\vec{b}$ symbolize two vectors of a point. θ equals to the space angle between two vectors and vector *C* is the parallel spatial line of $\vec{b}$. Therefore, the angle between $\vec{a}$ and *C* equals that of the $\vec{b}$ and *C*.

c. Cosine distance. It indicates the angular distance, namely, the cosine value of the angle between two eigenvectors. The larger the value is, the more similar the two eigenvectors are and vice versa.

### Expression Framework of HPE

**Figure 5:**
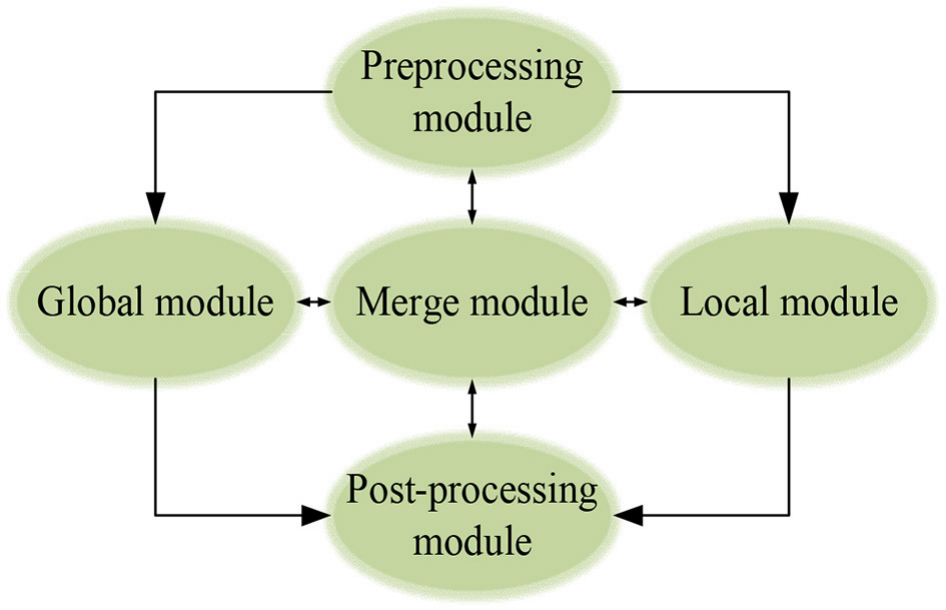
The modular expression framework.

Human Pose Estimation means estimating the coordinates and position of human joints relative to each part in a given image. Here, a single-lens camera is used for HPE. This section designs an expression framework to describe the whole image and express information-abundant local features effectively. Therefore, a rough human body model is implemented initially. Then, optimization is made on the local features in all directions. SWA can scan the whole image with pre-determined sliding window parameters in local feature modeling. A 220 × 220-pixel sliding window extracts the local features. Before a full-effect expression framework can be established, it is necessary to pre-process the input images so that DCNN can accept (Wang et al., 2019; Lv and Liang, 2020; Abid et al., 2021), as illuminated in Figure 5.

#### Pre-Processing and Post-Processing Module

The pre-processing module is used for data adjustment and enhancement during model training. In contrast, the post-processing module extracts' joint information of the athlete during training and competition and feedback the information.

#### Data Enhancement

Then, only minor changes need to be made to the existing data set to obtain more data, such as flipping, translation, or rotation. CNN can classify objects robustly even if they are placed in different directions. After translation, the viewpoint, size, or illumination (or a combination of the three) remain unchanged. Images collected from the internet will have different sizes. Since fully connected layers are fundamental design in most NNs, image size must be fixed before inputting the network. Popular data enhancement technologies operate pictures in the following ways: scaling, translation, rotation, flipping, adding noise, illumination adjustment, and perspective transformation.

Image recognition relies on BDT, and many training samples must be prepared. Accordingly, to weigh the shortage of training samples, the present work chooses to enhance the sample data, minimizing model overfitting or distortion from insufficient training. Then, a human body structure with *K* joints is expressed by Equation (3), where *L* will be used as the initial input of joints:


(3) $$\begin{array}{l} L=\left(l_{1},\cdot \cdot \cdot ,l_{i}\cdot \cdot \cdot \right),\left(i\in \left\{1,\cdot \cdot \cdot ,K\right\}\right) \end{array}$$

In (3), *l*_*i*_ = (*x*_*i*_, *y*_*i*_) is the coordinates of the *i*-th human joint.

Where *D* represents the position information, *L* is the actual value of human action. The original labeled sample is expressed as (*D, L*), which is expressed as $\left(D,\bar{L}\right)$ after the expression framework is adjusted. $D_{x}^{r}$ and $D_{y}^{r}$ denote the after-rotation abscissa and ordinate, respectively.

Data enhancement performs multiple operations on the original image. Then, to describe the coordinates after rotation, the top left corner of the image is set to be 0 point and its coordinates to be (1,1). From this point on, the Y-axis is set to top-to-bottom, the X-axis left-to-right. The picture size is (*D*_*x*_, *D*_*y*_). The rotation angle is φ; clockwise in the positive direction. ${l_{i}}^{r}=\left({x_{i}}^{r},{y_{i}}^{r}\right)$ is the coordinate of the rotated human joint *i*. *D*^*r*^ is the data of the rotated image.

- Rotation: as in Equations (4–7).

(4) $$\begin{array}{l} x_{i}^{r}=\left(x-\frac{D_{x}}{2}\right)\cos\varphi -\left(y-\frac{D_{y}}{2}\right)\sin\varphi +\frac{D_{x}^{r}}{2} \end{array}$$

(5) $$\begin{array}{l} y_{i}^{r}=\left(y-\frac{Dy}{2}\right)\cos\varphi -\left(x-\frac{Dx}{2}\right)\sin\varphi +\frac{D_{y}^{r}}{2} \end{array}$$

(6) $$\begin{array}{l} D_{x}^{r}=\left(D_{y}-D_{x}\tan\varphi \right)\sin\varphi +D_{x}\cos\varphi \end{array}$$

(7) $$\begin{array}{l} D_{y}^{r}=\left(D_{x}-D_{y}\tan\varphi \right)\sin\varphi +D_{y}\cos\varphi \end{array}$$

The rotation angle is used to obtain amounts of training samples randomly.

b. Translation: Different points on the image are selected to translate the human body, by which the minimum clipping area can be solved by Equations (8) and (9).
  The rotation angle is random to obtain many training samples.

(8) $$\begin{array}{l} C_{top-left}=(\min\left(x_{1}^{r},\cdot \cdot \cdot ,x_{K}^{r}\right),\min\left(y_{1}^{r}-y_{K}^{r}\right) \end{array}$$

(9) $$\begin{array}{l} C_{bottom-right}=(\left(\max\left(x_{1}^{r},\cdot \cdot \cdot ,x_{K}^{r}\right),\max\left(y_{1}^{r}-y_{K}^{r}\right)\right) \end{array}$$

Where *C*_*top*−*left*_ is the coordinate of the leftmost pixel in the clipping region, and *C*_*bottom*−*right*_ is at the bottom right. min and max represent the minimum and the maximum values in the sequence, $x_{K}^{r}$ and $y_{K}^{r}$ refer to the abscissa and ordinate after conversion.

c. Scaling
d. Horizontal turning: The coordinates after horizontal turning are expressed as Equations (10) and (11).

(10) $$\begin{array}{l} x_{j}^{f}=D_{x}^{S}-x_{i}^{S} \end{array}$$

(11) $$\begin{array}{l} y_{j}^{f}=y_{i}^{S} \end{array}$$

In Equations (10) and (11), $x_{j}^{f}$ refers to the inverted abscissa, $y_{j}^{f}$indicates the inverted ordinate, $D_{x}^{S}$ represents the data of the inverted image, $\left(x_{j}^{f},y_{j}^{f}\right)$ indicates the coordinate value of the inverted pixel, and *j* stands for the joint corresponding to the original joint *i*.

#### HPE and Evaluation

The Precision-Recall (P-R) curve is used to evaluate the detection effect. Equations (12) and (13) calculate the P and R values.


(12) $$\begin{array}{l} P=\frac{TP}{TP+FP} \end{array}$$

(13) $$\begin{array}{l} R=\frac{TP}{TP+FN} \end{array}$$

For a test, the relationship between the predicted value and the actual value has four situations as signified in the following: TP represents that the actual value of the positive sample is true, FP represents that the predicted value of the negative sample is true, TN represents that the predictive value of the positive sample is true, and FN represents that the prediction of negative sample is false. *P*indicates precision and *R* denotes recall.

### Deep Convolutional Neural Network

#### Deep Learning

In recent years, DL has been a relatively new and popular research direction in the ML field. DL enables computers to learn and summarize the internal laws of various data (sound, image, and other data) and finally power the computer to analyze and learn like humans. At present, many significant breakthroughs have been made in DL (Cocos and Fiks, 2019). At first, in 2006, DL appeared as a new research direction in ML and gradually been applied to various industries. In 2012, Stanford University took the lead in building a training model called DNN. Then, using 16,000 Central Processing Units (CPUs), DNN technology has made a great breakthrough in voice and IR applications. In 2016, alpha dog, an artificial go (Chinese “Weiqi”) software was developed based on DL, defeated Li Shishi, the top go game master in the world. Since then, many well-known high-tech companies worldwide have begun to invest tremendous material resources and talents in DL and setup relevant research institutes.

**Figure 6:**
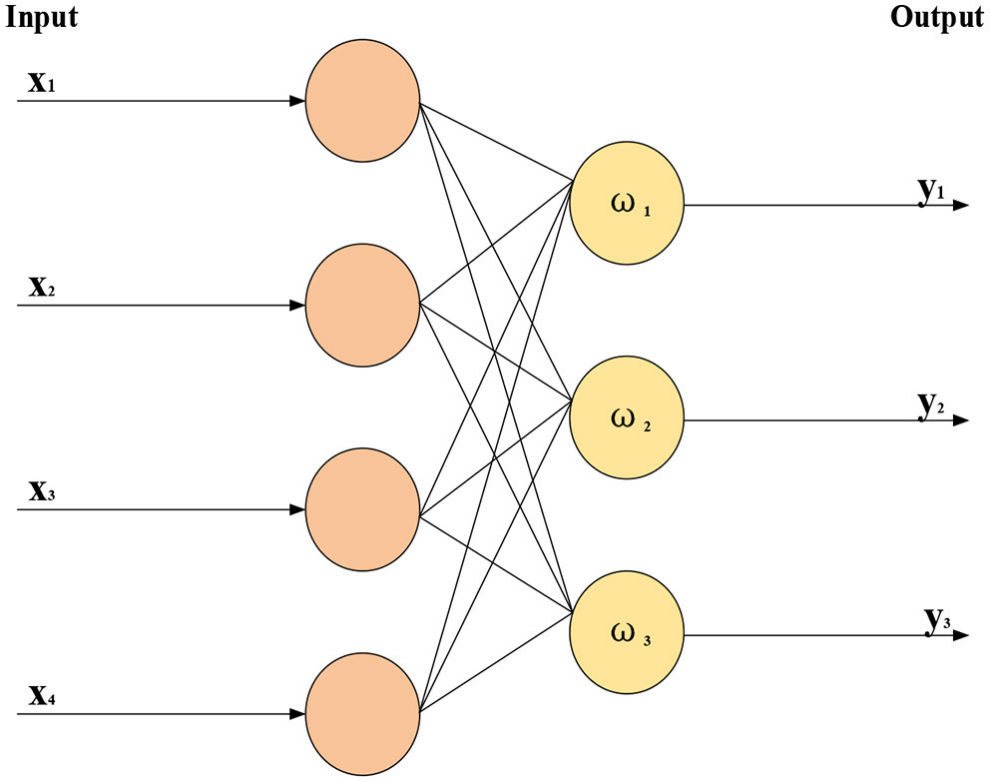
Structure of simple neural network.

Machine learning technology studies how computers simulate or realize learning behaviors of humans to learn new knowledge, rewrite existing data structures, and improve program performance. From a statistical perspective, ML is used to predict data distribution, model available data, and then predict new data, requiring testing and training data to conform to the same distribution. Its basic feature is to imitate the mode of information transmission and processing between neurons in the human brain. Computer vision and Natural Language Processing (NLP) are the most notable applications of ML. Obviously, “DL” is strongly related to “neural network” in ML. “Neural network” is also its main implementation algorithm and means. Thus, “DL” can also be called an “improved neural network” algorithm. The idea of DL is to simulate human neurons. Each neuron receives information and transmits it to all adjacent neurons after processing. Figure 6 sketches the structure of a simple NN.

#### AlexNet: A DCNN

The basic principle of AlexNet has been applied to many other deep network structures. The leading new technologies used by AlexNet are as follows:

- Rectified linear activation function (ReLU) is successfully used as the activation function (AF) of CNN, and its effect is verified to exceed that of Sigmoid function in deep NN. Thus, ReLU can solve the gradient dispersion problem of the Sigmoid function. Although the ReLU was proposed a long time ago, it has not seen further application until the emergence of AlexNet (Shorten and Khoshgoftaar, 2019).
- During training, Dropout is used to ignore some neurons randomly to avoid model overfitting. Dropout has been discussed in a separate paper, and AlexNet has put it into practice and proved its effect through practice. In AlexNet, Dropout is mainly used in several last fully connected layers.
- Overlapping maximum pooling (Max pooling) is introduced into CNN. Before AlexNet, CNN used average pooling. By comparison, AlexNet introduces Max pooling to avoid the blurring effect of average pooling. Additionally, AlexNet proposes to scale down the step size than the pooling core so that there will be overlap and coverage between the outputs of the pooling layer, which improves the richness of features.
- A local response normalization (LRN) layer is proposed to create a competition mechanism for local neurons. The value with a larger response becomes relatively larger and inhibits other neurons with minor feedback. As a result, it enhances the model generalization. In training many chain data, once the distribution of each batch of training data is different (batch gradient decline), the network must learn to adapt to different distributions in each iteration, which will significantly reduce the training speed.

**Figure 7:**
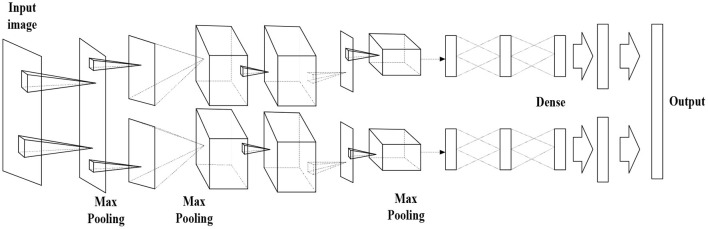
Deep Convolutional Neural Network (DCNN) structure.

Figure 7 displays the AlexNet-based DCNN structure, and Table 1 lists its detailed parameters.

**Table 1:** Detailed AlexNet-based Deep Convolutional Neural Network (DCNN) parameters.

Input image data (size: 227 *227 *3)		
Convolution layer 1	11 * 11 convolution kernel a, number = 48, step size = 4	11 * 11 convolution kernel b, number = 48, step size 4
	Activate function (relu)	Activate function (relu)
	Pooling layer (kernel size = 3, stride = 2)	Pooling layer (kernel size = 3, stride = 2)
	Standardization	
Convolution layer 2	Convolution layer size = 5*5, number = 128, step size = 1	Convolution layer size = 5*5, number = 128, step size = 1.
	Activate function (relu)	Activate function (relu)
	Pooling layer (kernel size = 3, stride = 2)	Pooling layer (kernel size =3, stride = 2)
	Standardization	
Convolution layer 3	Convolution kernel size = 3 * 3, number = 192, step size = 1	Convolution kernel size = 3 * 3, number = 192, step size 1.
	Activate function (relu)	Activate function (relu)
Convolution layer 4	Convolution kernel size = 3 * 3, number = 192, step size = 1	Convolution kernel size = 3 * 3, number = 192, step size 1.
	Activate function (relu)	Activate function (relu)
Convolution layer 5	Convolution kernel size = 3*3, number = 192, step size = 1	Convolution kernel size = 3*3, number = 192, step size = 1.
	Activate function (relu)	Activate function (relu)
	Pooling layer (kernel size = 3, stride = 2)	Pooling layer (kernel size = 3, stride = 2)
Fully connected layer 6	2,048 neurons	2,048 neurons.
	Dropout	Dropout
Fully connected layer 7	2,048 neurons	2,048 neurons.
	Dropout	Dropout
Fully connected layer 8	1,000 neurons	

The proposed AlexNet-based DCNN has five convolution layers, followed by the Max-pooling layer for down-sampling and three fully connected layers. The last layer is the SoftMax output layer, which has 1,000 nodes and corresponds to 1,000 image-classification options in ImageNet atlas. The network middle volume dividing base layer is calculated by two independent operations, which is beneficial to GPU parallelization and reduces calculation.

Two-dimensional (2D) CNN comprises a 2D convolution layer and a 2D pooling layer. Since the 2D CNN does not process object motion information in the time dimension between images, it cannot detect video sequences. Researchers proposed three-dimensional (3D) CNN to better extract video features by adding a time dimension to the convolution kernel (namely, the 3D convolution kernel) to obtain time-domain and spatial domain information. Thus, 3DCNN can achieve better recognition results. Three dimensional CNN forms a cube by stacking multiple consecutive frames. In this cube structure, each feature map in the convolution layer is connected to multiple adjacent consecutive frames in the previous layer to capture motion information.

**Figure 8:**
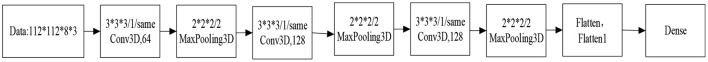
Structure of the improved three-dimensional Convolutional Neural Network (3DCNN).

The improved CNN model has three 3D convolution layers, three 3D pooling layers, one fully connected layer, one SoftMax layer, and two Flatten layers, as shown in Figure 8. The size of the 3D convolution kernel is [3 × 3 × 3], where “3 × 3” represents the spatial dimension, the third “3” is the time dimension, the step size is “1,” and padding is “1.” The number of convolution kernels from the first convolution layer to the sixth convolution layer is 64, 128, 256, 256, 512, and 512, respectively. Each convolution layer is connected to the pooling layer, and Max pooling is used throughout the network structure. The Max pooling can effectively eliminate the estimated mean shift caused by the parameter error of the convolution layer. The filter size of the first pooling layer is [1 × 2 × 2], and the size of the remaining filters is [2 × 2 × 2]. After the Dropout and Flatten layers, SoftMax is used to classify and get the class output. Figure 8 denotes an improved 3DCNN structure.

#### Multi-Instance Learning (MIL)

According to the ambiguity of training data, the research in this field can be roughly divided into three learning frameworks: supervised learning, unsupervised learning, and Reinforcement Learning (RL). Supervised learning marks the samples, while unsupervised learning does not need to mark them, so the learning model has great ambiguity. MIL can be considered the fourth learning framework juxtaposed with the three traditional learning frameworks. MIL can be described as follows: suppose that each datum in the training data set is a package, each package is a set of examples, each package has a training tag, and the examples in the package are tagless. Then, one positively marked example will make the whole package positive; all examples within a negatively tagged package are negative. Noticeably, here the tagging operation is in relation to the sample training. Differently put, the training operation only labels the package, not the examples, but the example labels do exist. Meanwhile, samples can be positive or negative and are used for further sample classification. All the sample tags are given in supervised learning but are unknown in MIL. By comparison, all examples are tagless in unsupervised learning, while the package in MIL is tagged. However, one characteristic of MIL is that it widely exists in the real world and has great potential application prospects.

#### Weakly Supervised Learning (WSL)

Supervised learning technology builds prediction models by learning many labeled training samples, which has succeeded in many fields. However, due to the high cost of data annotation, it is not easy to obtain strong supervision information, such as all truth labels, in many tasks. However, due to the lack of formulated labels, the performance of unsupervised learning in practical application is often very limited. To solve this problem, relevant researchers put forward the concept of WSL that can reduce the workload of manual marking and introduce supervised human information to improve the performance of unsupervised learning to a great extent.

Weakly Supervised Learning is relative to supervised learning. Unlike supervised learning, the data labels in WSL are allowed to be incomplete. That is, only part of the data in the training set has labels, and the rest or even most of the data are unlabeled. In other words, supervised learning is indirect. That is, the ML signal is not directly assigned to the model but indirectly transmitted to the ML model through some guiding information. In short, WSL covers a wide range. In some sense, tag learning can be regarded as WSL as long as the annotation information is incomplete, inaccurate, or imprecise. WSL mainly includes Semi-Supervised Learning (SSL), TL, and RL.

## Research Model and Framework

### Algorithm Model

**Figure 9:**
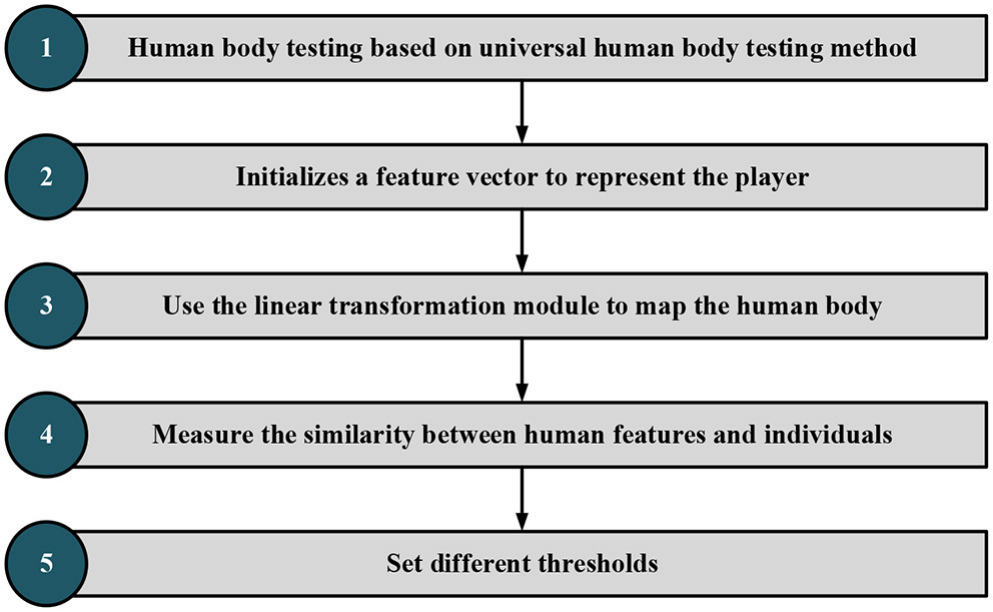
Identification and detection steps.

The recognition method based on the strength feature metric is used here. Figure 9 displays its recognition and detection steps. General human behavior recognition algorithm: spatio-temporal graph convolutional network (ST-GCN) refers to recognizing human behavior in the videos, namely, reading video. According to the types of actions and the processing tasks, the behavior recognition tasks in various cases are slightly different. First, compare and distinguish the two groups of concepts:

The steps of image content identification and detection are as follows:

The first step is to use the faster region CNN (FR-CNN) target detection model trained on Microsoft Common Objects in Context (MSCOCO) general dataset to detect human features. Part of the FR-CNN structure comes from the convolution layer of pre-training, and the other part is its unique structure. The training process is as follows: (a) initialize the network with the convolution layer of pre-training and carry out training. After training, the convolution layer of pre-training and its unique structure will be updated and (b) initialize the FR-CNN with the pre-training convolution layer. Note that the pre-training convolution layer is the same as the first step. The trained network is used to calculate the unique value, and then the updated suggestion is given to FR-CNN. Then, the model training continues to update the convolution layer of pre-training and the network structure of FR-CNN. In steps a and b, the FR-CNN is initialized with the same pre-training convolution layer and then trained independently. Therefore, the update of the pre-training convolution layer must be different after training, which means that the pre-training convolution layer is not shared. (c) The convolution layer pre-trained in step b is used to initialize the network and train the network for the second time. Notably, the convolution layer of pre-training should be locked this time. Differently put, the pre-trained convolution layer will remain unchanged during the training process. However, the unique structure will be changed, so the pre-training convolution layer will always be consistent with the pre-trained FR-CNN structure in step c. (d) Keep the pre-trained convolution layer in step c unchanged, initialize FR-CNN, and train FR-CNN for the second time.

In the second step, an initial vector represents main trunk of the athlete, and the SWA is used for FE. A rectangular box with multiple scales is specified in advance. The sliding window slides from left to right and from top to bottom according to a particular step from the upper left corner of the image. Each sliding position is reserved as a bounding box. Increasing the rectangular box type and reducing the sliding step allow the bounding box to put down all detected objects. The linear transformation module is trained by the MIL method. When it is known that the image contains object categories, the candidate frame of the image is extracted first. These bounding boxes form a negative package for an image that does not contain a target object. These bounding boxes form a positive package for an image containing a target object. The goal of MIL is to determine which bounding box in the positive package is the target object. After continuous MIL between positive and negative packets, the category of bounding boxes is finally determined to complete object localization. There is no need to label the position and category of the boundary.

In the third step, the linear transformation module represents the human trunk features. The feature is extracted on the feature map of the target detection model according to the human trunk detection frame, and the linear transformation module is trained in the form of WSL. In this paper, RL is selected, a typical WSL algorithm. Unlike supervised learning, RL needs to try to find the results of each pose, and there is no training data to tell the machine which pose to estimate. However, an appropriate reward function can make the ML model learn the corresponding strategies under the guidance of the reward function. The goal of RL is to study how to learn a behavior strategy to maximize the cumulative reward in interaction with the environment. In short, RL keeps trying in the training process, deducting points if the decisions are wrong and reward points if otherwise to get the best decision in each state environment.

The fourth step is to map human features into the feature space of the athlete to measure the similarity between human features and athlete features. First, the video is segmented into frames and then the general target detector based on FR-CNN is used to detect the people in each frame. Afterward, the detection results of the athletes are distinguished by the trained example feature measurement model. Then, the multi-frame detection result fusion strategy based on video time-domain context is used to optimize the detection results of each frame. Further, according to the detection frame of the athlete, the image is enlarged, cut to a certain extent, and sent to the single-person pose detector. Finally, the detection results of the single HPE module are mapped back to the original image to obtain the complete athlete-oriented HPE of each frame.

Fifth, to effectively distinguish whether there is inter-frame mutation and local mutation, it is necessary to calculate the inter-frame similarity and local similarity and set the threshold to distinguish. Meanwhile, to calculate the similarity of images, there is a need to extract the image features. Specifically, this paper adopts the Haar-like feature method to compare positions of athletes between multiple frames. The threshold is set to distinguish athletes from non-athletes, and the detection results are optimized based on the video time-domain context information.

As in Figure 9, image frames with “someone” are classified as positive packets and frames without “someone” as negative packets. The detection frame extracts the image features that are then transformed into the same domain space. The maximum feature similarity is taken as the positive packet similarity. Following a similar procedure, the negative packet similarity is determined. Figure 9 draws the target detection frame of the algorithm (Ma et al., 2019; Afrasiabi et al., 2021).

### Description of Experimental Data Sets

**Figure 10:**
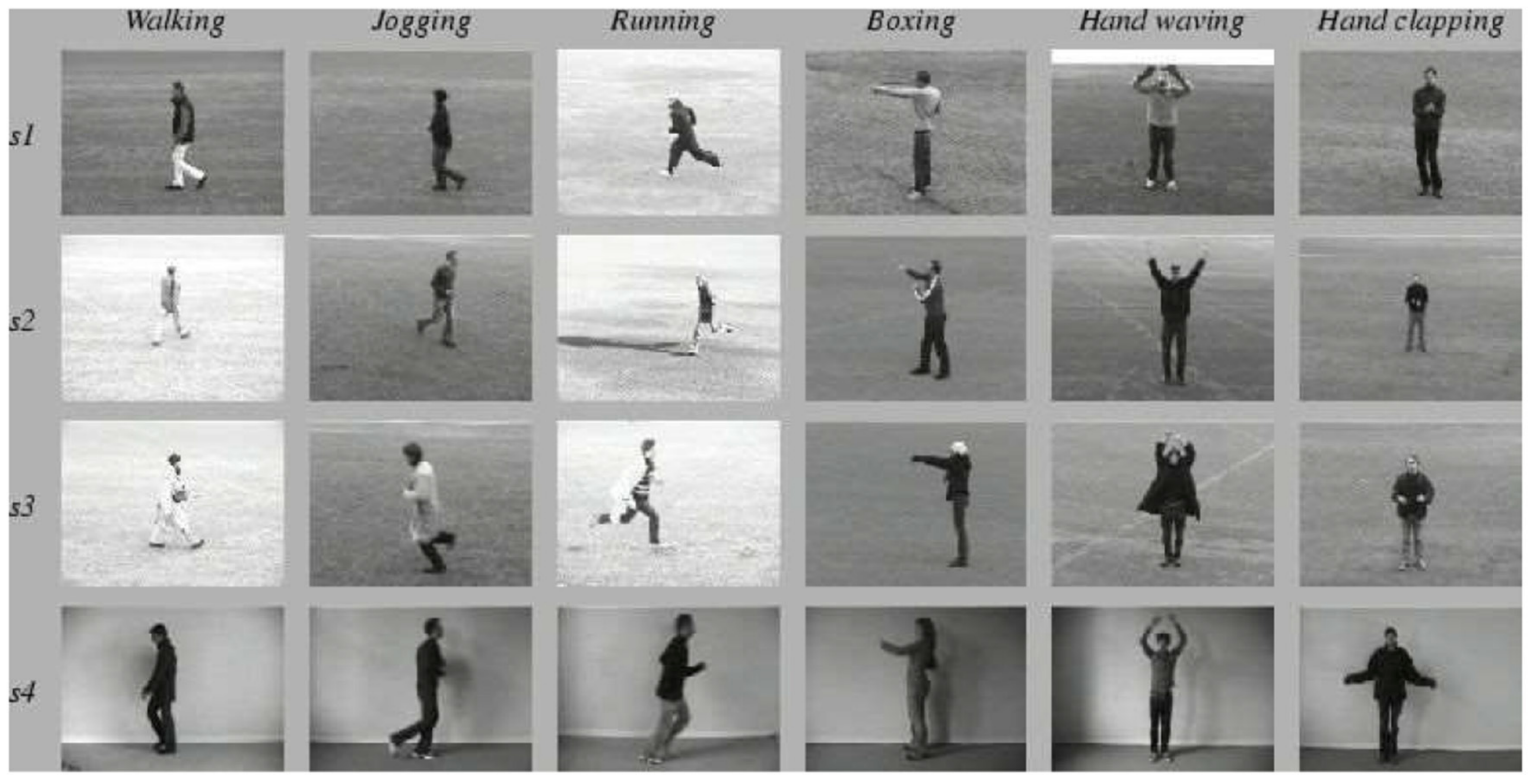
KTH datasets.

KTH dataset was released in 2004, which contains six types of human behaviors: walking, jogging, running, boxing, waving, and applause. Each type of behavior is performed by 25 people in four different scenarios (outdoors, outdoors with scale changes, outdoors with clothing changes, and indoors) multiple times, fixed with cameras. The database has a total of 2,391 video samples with 25 Frames Per Second (FPS), a 160 × 120 resolution, and a 4-s average length, as portrayed in Figure 10.

**Figure 11:**
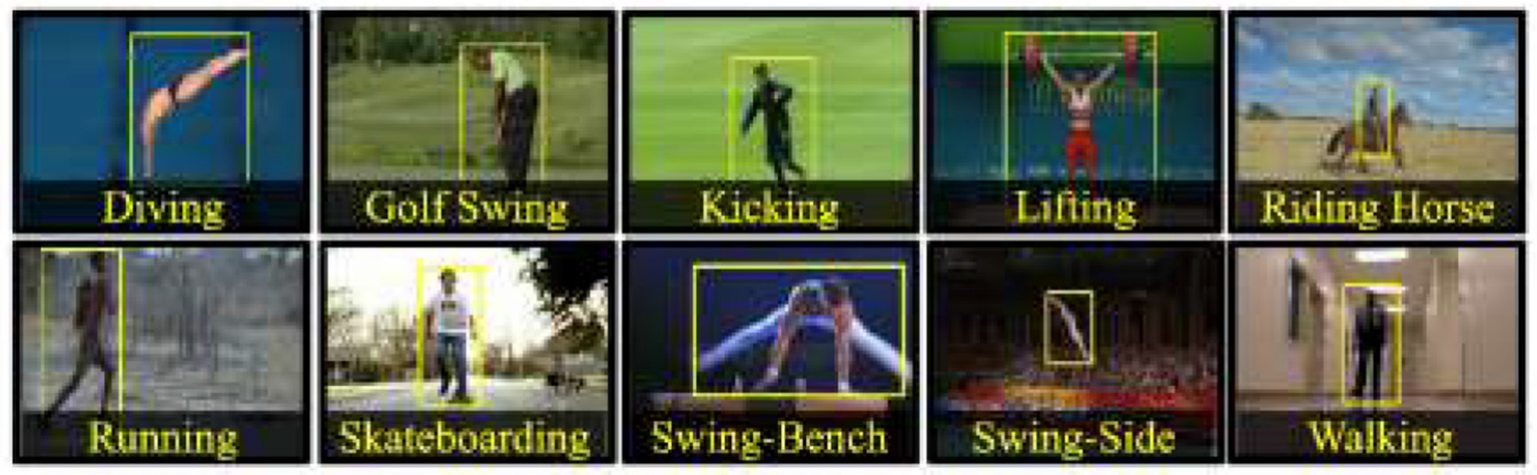
UCF Sports datasets.

**Figure 12:**
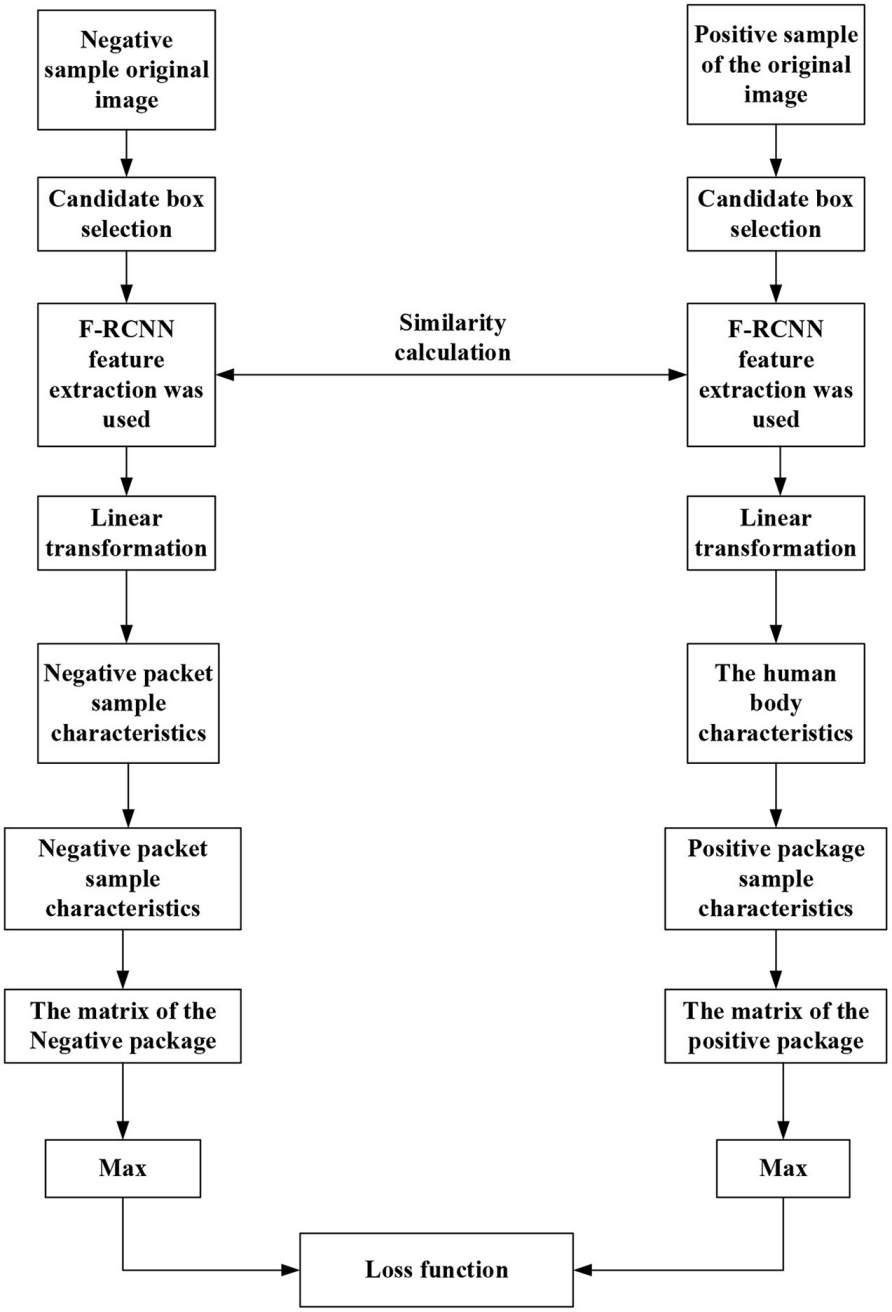
Target detection frame of algorithm.

The UCF Sports action library is mainly composed of 13 conventional sports actions. This data set consists of a series of actions collected from various sports activities, which are usually broadcast on TV channels, such as British Broadcasting Corporation (BBC) and Entertainment and Sports Programming Network (ESPN) (Kumar et al., 2021). These video sequences are obtained from various material websites, as outlined in Figure 11. Figure 12 shows the target frame detection algorithm.

### Preparation of Simulation Experiment

The single-stage-based YOLOv3 detection algorithm model, two-stage-based FR-CNN, Anchor-free, CornerNet, and Merge Soring algorithm are selected as the control group. The model recognition is tested on the MSCOCO dataset, and the largest feature map is selected for FE. Finally, experiments are carried out using the extracted football video and volleyball training video data.

Experiment 1: Different diving training videos of a sports college are collected as the experimental objects, with a resolution of 342 × 2,200.

Experiment 2: The database uses the KTH action library and UCF Sports action library. The experiment uses the proposed method to identify the confusion matrix of the two experimental action libraries. The KTH library trains the models using 35 athletes' sports training videos, tests the effect of the model on one person for 36 rounds of cross-detection, and averages the accuracy of pose recognition. Each type of video in the UCF Sports database is randomly selected as a testing video. The remaining video is used as a training video for 50 rounds of cross-validation. The pose recognition accuracy is averaged.

Experiment 3: The proposed performance of the model is compared to the top-down athlete-oriented HPE model based on AlphaPose, the bottom-up athlete-oriented HPE model based on PifPaf, and the athlete-oriented HPE model based on PifPaf with local spatial constraints. Then, 300 football and volleyball training video frames are extracted to generate a training set with 200 pictures and a test set with 100 pictures, respectively. Altogether, 600 non-athlete images are extracted and clustered into a training set that contains 400 images and a testing set that contains 200 images (Wang et al., 2021).

## Experimental Design and Performance Evaluation

### The Effectiveness of Feature Metrics for Detection Results

**Figure 13:**
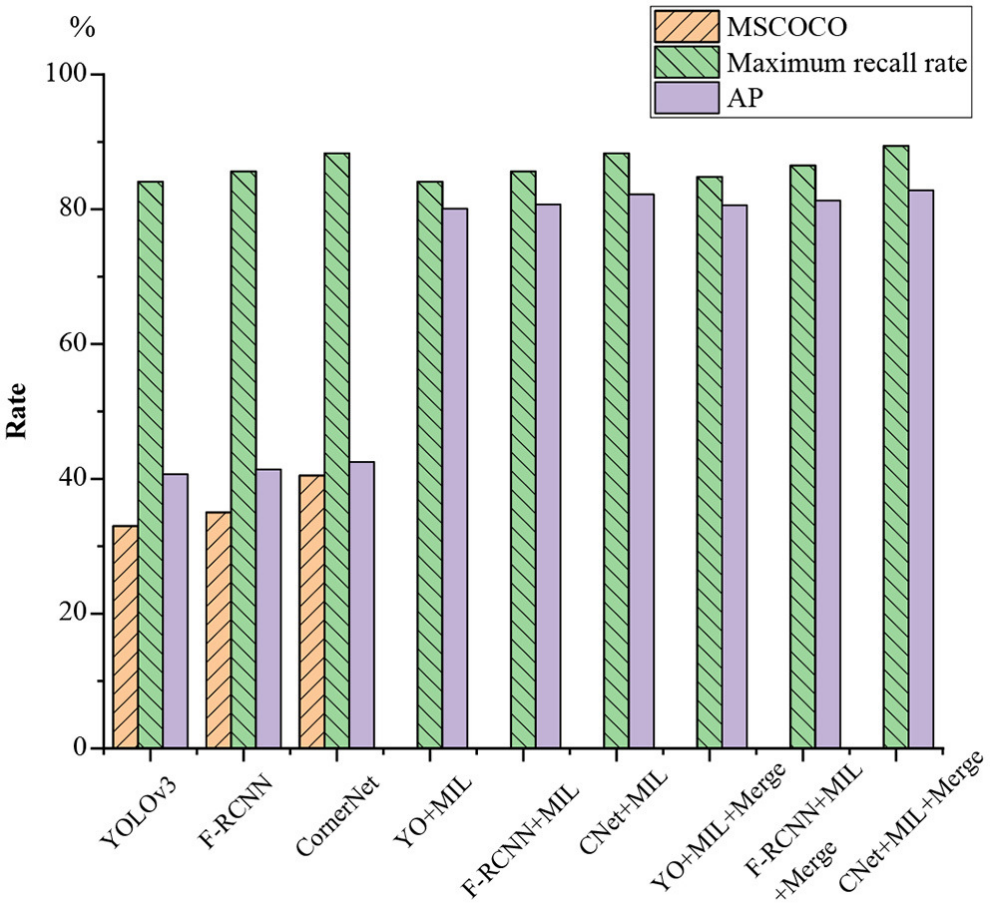
Football video detection results.

**Figure 14:**
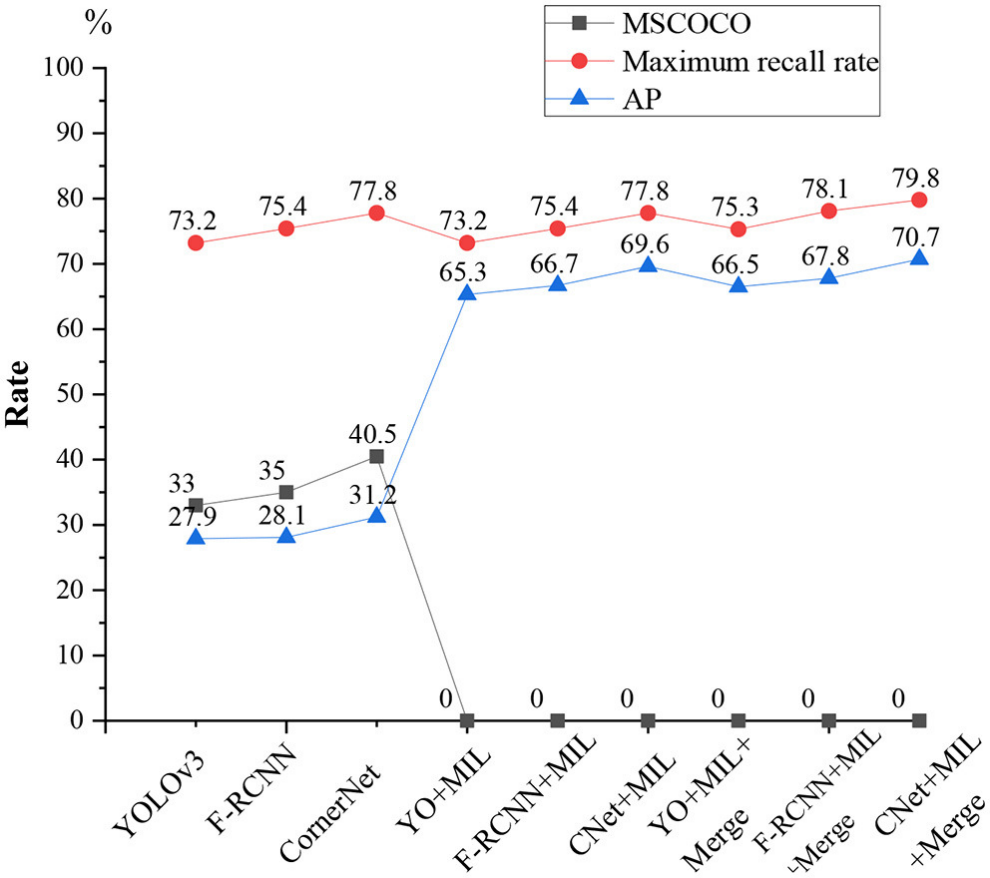
Volleyball video detection results.

The proposed validity of the algorithm is tested on the football and volleyball video datasets through comparative analysis with YOLOv3, F-RCNN, CornerNet, YOLOv3 + the proposed algorithm, F-RCNN + the proposed algorithm, CornerNet + the proposed algorithm, YOLOv3 + the proposed algorithm + Merge, and CornerNet + the proposed algorithm + Merge. Figures 13, 14 plot the recognition accuracy, maximum recall, and accuracy of several models on the MSCOCO dataset.

As in Figures 13, 14, although there are differences in the effectiveness of various models on different motion data sets, the differences are relatively low in HPE accuracy, and the maximum recall differences are the lowest. Additionally, the maximum recall of individual YOLOv3, FR-CNN, the Anchor-free, and CornerNet algorithms is normal. However, the average accuracy on MSCOCO and AP is only 20–40%, so using a general detection algorithm alone for sports video detection and recognition is not feasible. However, the average accuracy is greatly improved when the feature metric detection method is added to the general model; the maximum recall and average accuracy are both improved to over 65%. The maximum recall of football videos is over 80%, while the maximum recall is generally over 70% for volleyball videos.

### Performance Comparison Experiment

**Figure 15:**
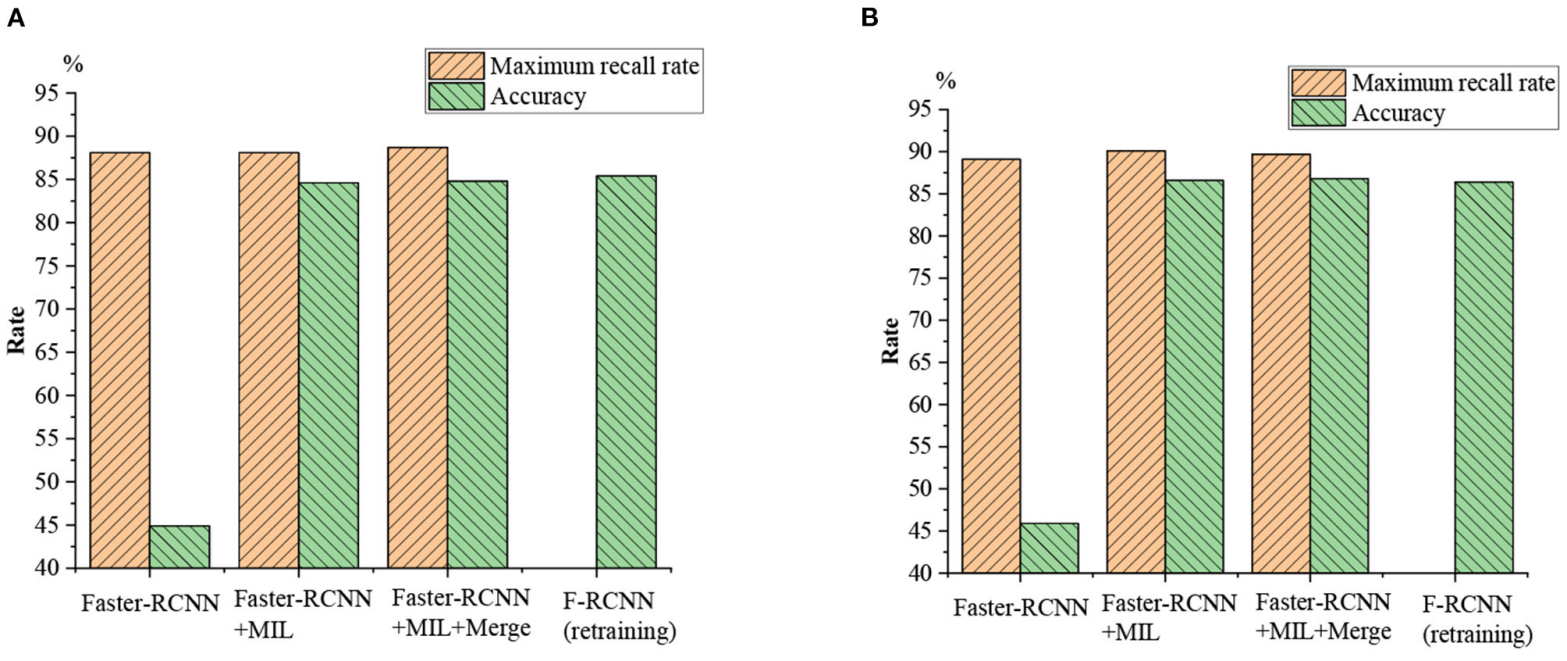
Comparative experiment of mixed sports video recognition. **(A)** Retraining rate as 0.01; **(B)** retraining rate as 0.001.

Different kinds of mixed sports videos are used for identification and contrast experiments, and the results are displayed in Figure 15. The performance of the proposed athlete-oriented HPE model is compared with that of the mainstream HPE model, as plotted in Table 2.

**Table 2:** Performance comparison of the same Human Pose Estimation (HPE) algorithm.

**Model**	**Deep**	**Joint**	**Athlete**	**Complete**	**Accuracy**
**name**	**network**	**point**	**pose**	**test**	
	**computing**	**matching**	**matching**	**time**	
	**time**	**time**	**time**		
FR-CNN	147 ms		10 ms	157 ms	86.1%
Alphapose	158 ms			203 ms	78.3%
Pifpaf	258 ms	102 ms	10 ms	443 ms	79.5%
Local Pifpaf	213 ms	302 ms		294 ms	79.6%

Figure 15 suggests that the FR-CNN model algorithm shows higher recognition accuracy on mixed sports videos than on a single sports video dataset, which may be related to the previously established data set. After retraining, the recognition accuracy of the FR-CNN algorithm has increased from <45–85%, with significant improvement. The maximum recall for the other two matching algorithms is not much different from that of a single FR-CNN model algorithm. In comparison, the recognition accuracy of the FR-CNN model algorithm before and after retraining with the mixed sports videos is not much different (from 40% to a bit higher). Therefore, the proposed athlete-oriented HPE algorithm has presented the best maximum recall and accuracy.

**Figure 16:**
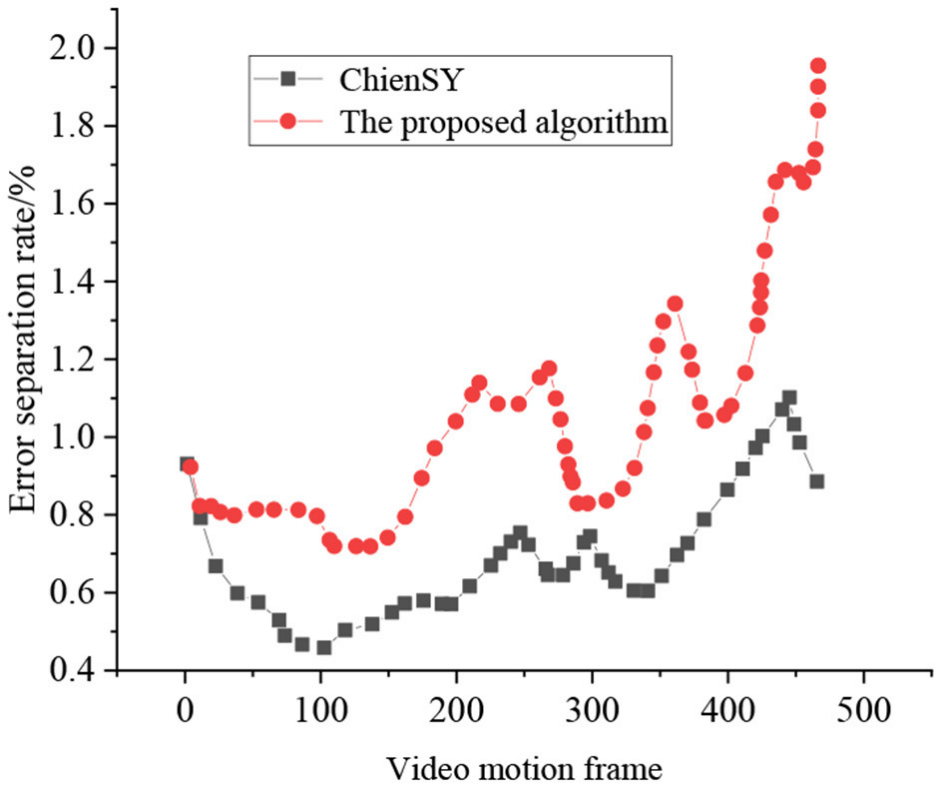
Test results of false separation rate.

Figure 16 signifies that the separation results obtained by the ChienSY method have obvious cavity problems, and the results obtained by the proposed HPE algorithm are complete and transparent. The false separation rate of the proposed method is about 1%, while that of the ChienSY method is about 3.4%. Due to biased parameter interference in the camera static motion model, the error separation rate of the ChienSY method shows a significant increasing trend in the later stage. In contrast, the error separation rate of the proposed algorithm does not show significant changes, indicating that the proposed method can accurately separate sports videos.

**Figure 17:**
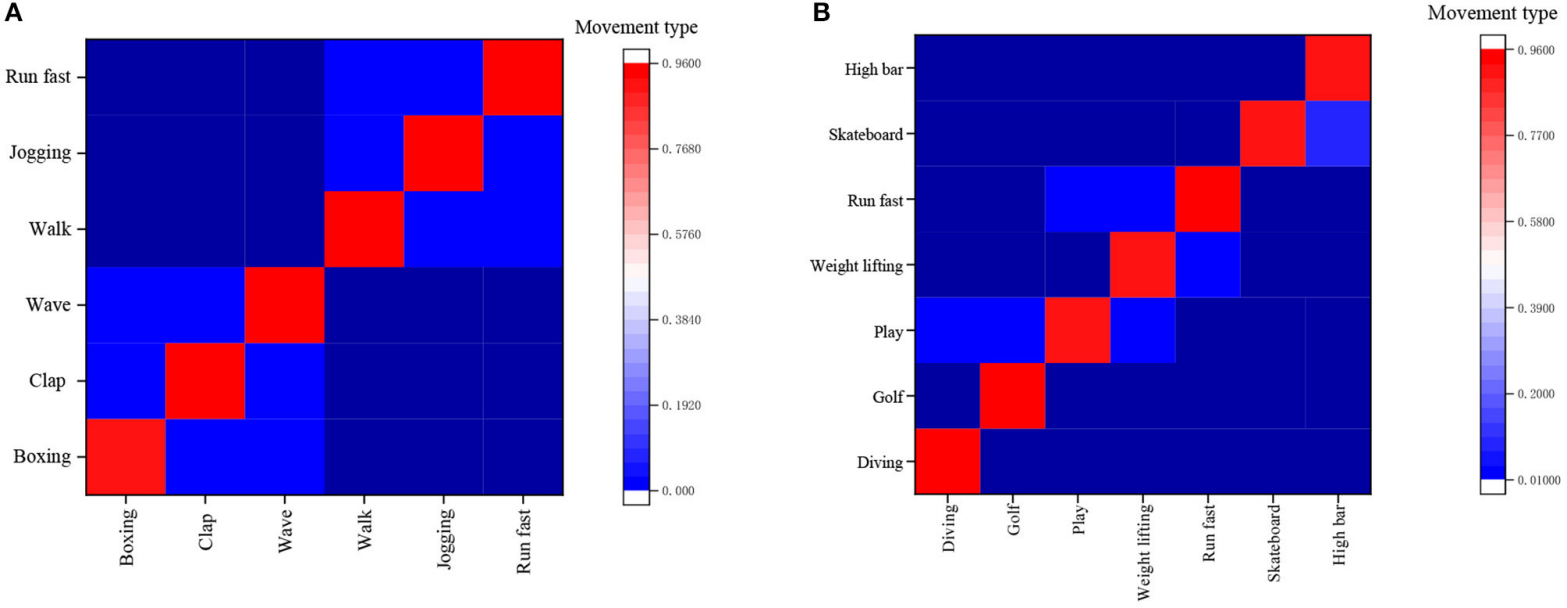
Recognition results of the confusion matrix. **(A)** Results of KTH confusion matrix, **(B)** Results of UCF Sports confusion matrix.

Figure 17A gives the identification results of the KTH confusion matrix by the proposed method, and Figure 17B presents the identification results of the UCF Sports confusion matrix by the proposed method.

Figure 17A concludes that the proposed method can separate the first and last three types of actions in the KTH database. Still, the recognition accuracy for walking, jogging, and fast running is relatively low, mainly because these three human motions are hard to discern from one another in terms of the involved body joints' motion amplitude. Figure 17B implies that it is basically the same as the recognition accuracy of various actions in the confusion table of the UCF Sports database. It is low of the variance of recognition accuracy of various actions in the database. Table 2 corroborates that the athlete-oriented HPE model's estimation time is mostly wasted on the NN, while no time has been spent on some modules. This is because the sensitivity of the research experiment is millisecond (ms), and the time span <1 ms is all denoted as 0 ms. In conclusion, the proposed FR-CNN algorithm has shown the fastest detection speed, as small as 157 ms, and its accuracy is much higher than the other three mainstream models, reaching 86.1%.

## Conclusion

Combined with BDT and DCNN algorithm, this study proposes an athlete-oriented FR-CNN-based HPE model to automatically recognize human actions and sports types from sports videos and finally realize sports video classification and hot spot detection. Concretely, BDT is mainly used to search the training set and testing set of sports video data. Then, the HPE model is realized by the DCNN algorithm, and the feature recognition algorithm is established to recognize the motion of the athlete. Finally, the proposed HPE model is trained with mixed motion videos. Final experiments demonstrate that by combining BDT with DCNN algorithms, the proposed HPE model effectively recognizes human motions in sports videos and can score accurately.

Although the expected objectives have been achieved, there are still some limitations. The compatibility of the model is insufficient, so future research work will focus on further improving the compatibility of the model and optimizing the code. (2) Due to the inability to obtain complete television and network video ratings and program information, the model may not accurately identify complex motion movements. Therefore, future research work will focus on further collecting relevant data, constructing reasonable data sets, understanding the actual situation and collecting data, and constructing data sets to optimize the model.

## Data Availability Statement

The raw data supporting the conclusions of this article will be made available by the authors, without undue reservation.

## Ethics Statement

The studies involving human participants were reviewed and approved by Beijing Sport University Ethics Committee. The patients/participants provided their written informed consent to participate in this study. Written informed consent was obtained from the individual(s) for the publication of any potentially identifiable images or data included in this article.

## Author Contributions

All authors listed have made a substantial, direct, and intellectual contribution to the work and approved it for publication.

## Conflict of Interest

The authors declare that the research was conducted in the absence of any commercial or financial relationships that could be construed as a potential conflict of interest.

## Publisher's Note

All claims expressed in this article are solely those of the authors and do not necessarily represent those of their affiliated organizations, or those of the publisher, the editors and the reviewers. Any product that may be evaluated in this article, or claim that may be made by its manufacturer, is not guaranteed or endorsed by the publisher.
